# Hydrastis Canadensis

**Published:** 1891-04-04

**Authors:** 


### THERAPEUTICS.

### HYDRASTIS CANADENSIS.

The Hydrastis Canadensis, or Golden Seal, has long been used by the North American Indians for a yellow dye extracted from the root, the part of the plant used in medicine. The rhizome, when fresh cut, is bright yellow ; this, when dried, assumes a dark yellowish-brown colour. It has a bitter and peculiar taste. The active principle, hydrastin, was first discovered by Mr. A. B. Durand, of Philadelphia. A second alkaloid, berberine, which imparts the yellow colour to the root, was identified by Mr. F. Mahla as berberine, an alkaloid not peculiar to this plant. Hydrastin forms brilliant colourless crystals in four-sided prisms, without smell, and with hardly any taste, being insoluble in saliva. It is almost insoluble in water, but is dissolved by ether, alcohol, or chloroform. Its physiological action is mainly on the spinal cord, producing violent tetanic spasms, the fatal result being due to asphyxia from spasm of the respiratory muscles. Fellner has shown that its action is accompanied by a slight rise in arterial pressure, large doses lowering arterial pressure. No rise of arterial pressure followed if the spinal cord was previously divided, thus its action in this respect is probably due to its stimulation of the vaso-motor centre, the larger doses exhausting and paralysing this centre, and thus producing a fall in the arterial pressure. Fellner and others have observed its powerful action on the uterus, uterine contractions being produced in the non-pregnant uterus, and in pregnant rabbits abortion. Rutherford has found it a stimulant of the hepatic secretions. In its local action on the mucous membranes it has proved of considerable service. It has been successfully applied in catarrh, especially in the gastro-intestinal catarrh of alcoholics. It has also been used in chronic pharyngitis, otorrhœa, nasal, urethral, and vaginal catarrh, and in the later stages of gonorrhœa. Internally administered, it is of greatest service in various uterine disorders, especially in congestive dysmenorrhœa, and in uterine myomata. A new alkaloid has recently been prepared by Dr. Falk, to which he gives the name hydrastinine. It is obtained by heating a mixture of hydrastin and nitric acid, and precipitating with an alkali. Hydrastinine is a white crystalline powder. The most convenient salt for mixtures appears to be the hydrochlorate, which is freely soluble in water. Its physiological action is not identical with that of hydrastin, it having a more decidedly stimulant action in the vaso-motor centres, producing a more persistent contraction of the vessels, without giving rise to the same degree of spinal irritation, and not producing tetanic convulsions in fairly large doses. This drug has been recommended by Dr. Falk, after considerable experience, as a uterine hœmostatic, preferable to ergot and ergotine. He injects a five to ten per cent. solution hypodermically, and in none of his cases did he observe the slightest irritation at the seat of the injection. Dr. Falk's experience with hydrastinine is not borne out by some other observers, who have found it less powerful in its action as a hœmostatic than hydrastin.

